# A randomized controlled trial of Mindfulness-Based Cognitive Therapy (MBCT) versus treatment-as-usual (TAU) for chronic, treatment-resistant depression: study protocol

**DOI:** 10.1186/s12888-015-0647-y

**Published:** 2015-11-09

**Authors:** Mira B. Cladder-Micus, Janna N. Vrijsen, Eni S. Becker, Rogier Donders, Jan Spijker, Anne E. M. Speckens

**Affiliations:** Behavioural Science Institute, Radboud University, Nijmegen, The Netherlands; Pro Persona Expertisecentrum Depressie, Institute for Mental Health Care, Nijmegen, The Netherlands; Department of Psychiatry, Radboud university medical centre, Nijmegen, The Netherlands; Department for Health Evidence, Radboud university medical centre, Nijmegen, The Netherlands

**Keywords:** Mindfulness-Based cognitive therapy, Chronic depression, Treatment-resistant depression, Randomized-controlled trial, Depressive symptoms, Rumination, Quality of life

## Abstract

**Background:**

Major depression is a common psychiatric disorder, frequently taking a chronic course. Despite provision of evidence-based treatments, including antidepressant medication and psychological treatments like cognitive behavioral therapy or interpersonal therapy, a substantial amount of patients do not recover. Mindfulness-Based Cognitive Therapy (MBCT) has been found to be effective in reducing relapse in recurrent depression, as well as lowering symptom levels in acute depression. The effectiveness of MBCT for chronic, treatment-resistant depression has only be studied in a few pilot trials. A large randomized controlled trial is necessary to examine the effectiveness of MBCT in reducing depressive symptoms in chronic, treatment-resistant depression.

**Methods/Design:**

A randomized-controlled trial is conducted to compare MBCT with treatment-as-usual (TAU). Patients with chronic, treatment-resistant depression who have received antidepressant medication and cognitive behavioral therapy or interpersonal therapy are included. Assessments take place at baseline and post intervention/TAU-period. The primary outcome are depressive symptoms. Secondary outcomes are: remission rates, quality of life, rumination, mindfulness skills and self-compassion. Patients in the TAU condition are offered to participate in the MBCT after the post TAU-period assessment. From all completers of the MBCT (MBCT condition and patients participating after the TAU-period), follow-up assessments are taken at three and six months after the completion of the MBCT.

**Discussion:**

This trial will result in valuable information about the effectiveness of MBCT in chronic, treatment-resistant depressed patients who previously received antidepressant medication and psychological treatment.

**Trial registration:**

trialregister.nl NTR4843, registered 14^th^ October 2014.

## Background

Major depression is one of the most common psychiatric disorders. It has a large impact on individual’s wellbeing and leads to high societal costs [1, 2]. The duration of a depressive episode is highly variable. Whereas the majority of people in the general population with a major depressive episode recovers within several months, approximately 20 % of all depressed patients develop a chronic course of depression [2, 3]. The latter is associated with higher suicidality [4], increased comorbidity with other psychiatric disorders, and impaired physical and psychosocial functioning [5]. According to DSM-IV criteria, a depressive episode is defined as chronic if the depressive episode persists for at least two years [6]. However, results of a large prospective epidemiological study on the prevalence of major depression in the Dutch population show, that -even if treated in mental health care- the rate of recovery decreases after a few months. After one year, recovery rates virtually stagnate, meaning that if no recovery has taken place within 12 months the probability to develop a chronic course of depression- as defined in the DSM-IV- increases substantially [3].

Not only a chronic course of symptoms but also several unsuccessful therapeutic interventions can be indicative of a severe depressive episode. Patients who do not respond to treatment are often described as ‘treatment-resistant’. However, up till now, an unified and clear definition of ‘treatment-resistant depression’ is lacking. Definitions range from one single unsuccessful attempt at antidepressant medication (ADM) to several pharmacological interventions combined with electro-convulsive therapy [7, 8]. In most clinical studies, a depressive episode is regarded as treatment-resistant when successive treatment with two antidepressants has not led to a significant improvement of depressive symptoms. Interestingly, this definition does not take response to psychological treatment into account, while cognitive behavior therapy (CBT) or interpersonal therapy (IPT) are part of international treatment guidelines for recurrent and chronic depression [9]. Importantly, research has shown that even when psychological and pharmacological treatment options are combined, a substantial amount of patients fails to respond [10, 11]. This means that there is a dire need of new treatment options for chronic, treatment resistant depression.

In recent years, mindfulness based interventions have been developed as a treatment focused on distress caused by long-lasting somatic and psychological disorders [12]. Mindfulness is defined as non-judgmental awareness of the present moment [13] and was introduced by Kabat-Zinn [14] as a treatment option for patients with chronic somatic conditions. Segal, Williams and Teasdale [15] adapted the program for patients with recurrent depression, naming it ‘Mindfulness Based Cognitive Therapy’ (MBCT). MBCT is a group intervention, consisting of eight weekly 2.5 h sessions and a silent day of 6 h. It involves meditation exercises as well as cognitive behavioral techniques. MBCT is designed to address processes that are associated with the onset, maintenance and recurrence of depression, such as maladaptive automatic cognitive and behavioral patterns in reaction to negative emotions or thoughts. In first instance participants are taught to allow and acknowledge negative emotions and thoughts as they are, without having judgments about them or reacting to them. In this way, they start recognizing their own maladaptive automatic patterns. By creating a greater awareness of habitual behavioral and cognitive patterns (e.g. avoidance or rumination) participants learn to deliberately disengage from them. Another important part of MBCT is to develop self-compassion. MBCT differs from CBT in the sense that the focus of cognitive therapy is more on the content of thoughts, while MBCT focuses on the process of thinking.

In chronic depression, maladaptive automatic cognitive and behavioral patterns often have become habitual. Patients often experienced several episodes of depression and an early onset of the disorder. To learn to react in a different way to these maladaptive automatic cognitive and behavioral patterns and to develop a non-judgmental awareness of depressive symptoms might be especially useful for this population. Therefore MBCT might be effective in lowering depressive symptoms in chronic, treatment resistant depression.

A recent meta-analysis [16] shows that MBCT is effective in preventing relapse in patients with recurrent depression. Additionally, there is evidence, that MBCT not only decreases chance of relapse of depression, but that it lowers levels of residual or current depressive symptoms. The first studies investigating the effect of MBCT on residual symptoms in remitted depressed patients found significant improvements of depressive symptoms [17, 18]. More recently, a meta-analysis was conducted on the effects of MBCT in patients currently meeting diagnostic criteria for a major depressive disorder [19]. The authors concluded that MBCT reduces depressive symptoms in currently depressed patients and might be as effective as CBT. One of the studies [20] included in the meta-analysis specifically investigated possible differences of the effectiveness of MBCT between remitted but still symptomatic and currently depressed patients. The results indicate that MBCT was effective for both remitted and currently depressed patients.

First results indicate that MBCT might also be a promising treatment option for chronic depression. Kenny and Williams [21] reported a decrease in depressive symptoms in a sample of currently depressed patients who experienced symptoms for one year or longer, preceded by three or more previous episodes. In line with that, Eisendrath et al. [22] found a significant decrease in depressive symptoms after MBCT in currently depressed patients who previously received at least two different antidepressants before starting the MBCT training. In addition to these non-randomized studies, two randomized-controlled pilot trials demonstrated a significant reduction of depressive symptoms in chronically depressed patients. Barnhofer et al. [23] found that after following MBCT less participants met full criteria for a major depressive episode compared to a control group. Another study [24] investigated the effectiveness of person-based cognitive therapy, a therapy combining mindfulness techniques and cognitive therapy, in chronic depression and found significant improvement of depressive symptoms and mindfulness skills in the therapy group compared to the control group. Importantly, all of these studies report a good acceptance of the MBCT training procedures by chronically depressed patients.

In sum, there is preliminary evidence that MBCT might be effective in reducing depressive symptoms in chronic, treatment-resistant patients. However well-powered randomized controlled trials are necessary to systematically examine the effectiveness of MBCT compared to treatment as usual in this severely affected patient group. With the current ongoing trial we will hopefully contribute to the knowledge about treatment options for patients with persistent depressive symptoms. Taking into account the high risk for chronic depression if symptoms persist for more than one year [3] we will recruit patients experiencing a depressive episode for 12 months or longer who underwent previous pharmacological and psychological treatment.

### Aims

The aim of the current study is to investigate the effect of MBCT compared to treatment as usual (TAU) on depressive symptoms in patients with a current depressive episode persisting for at least 12 months, who previously received pharmacological treatment, as well as either CBT or IPT. We hypothesize that patients who receive MBCT in addition to TAU will report lower levels of depressive symptoms, higher quality of life, and less rumination, compared to TAU alone. In addition, we expect that mindfulness skills and self-compassion will improve in the MBCT condition. Possible effects of level of treatment-resistance and experience of childhood trauma on treatment effect will also be investigated.

## Methods/Design

### Design

**Fig. 1:**
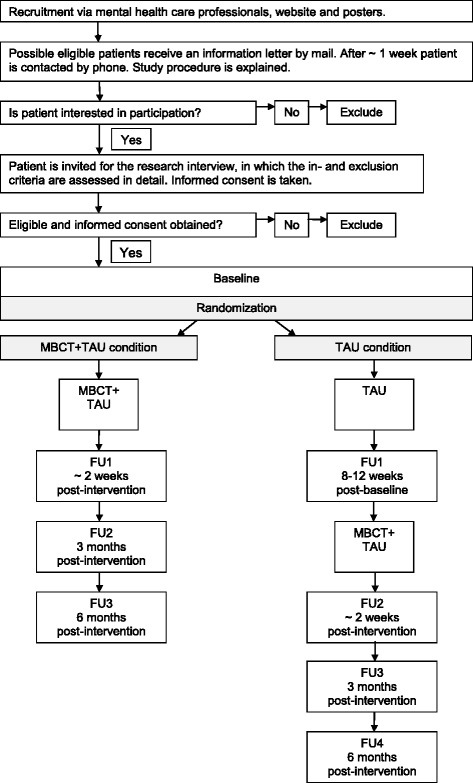
Flowchart of the recruitment and measurement points

This study is a multi-center open-labeled randomized controlled trial with two groups: MBCT+ TAU vs. TAU. The study has been approved by the Medical Ethics Committee Arnhem-Nijmegen (nr. 2012/339) for all participating sites. Before randomization, written informed consent is obtained from all participants. Primary and secondary outcome measures are assessed at baseline and after MBCT + TAU (8–12 weeks) or after a TAU-period (8–12 weeks). Participants allocated to the TAU condition are offered participation in the MBCT after completion of the TAU-period and first follow-up assessment. Of all participants who completed the MBCT, follow-up measures take place at three and six months after the end of treatment. For an overview of the recruitment and measurement points see Fig. 1.

### Participants

The study population consist of outpatients with a current depressive episode lasting for 12 months or longer who previously received ADM, as well as either CBT or IPT. Patients are recruited at a university medical center (Radboudumc Centre for Mindfulness) and at different locations of a regional mental health institution (Pro Persona) in The Netherlands. Patients can be referred by health care professionals, or can contact the research team themselves for participation. Patients receive an information letter and are contacted by phone one week later by the research team. Patients are informed that participation in the study is completely voluntary and that they can withdraw from the study at any time. Participants are free to receive additional treatment or to change their (dose of) pharmacological medication during the study period.

Inclusion criteria:

- Age ≥ 18
- A current diagnosis of major depressive disorder according to DSM-IV criteria with a duration of ≥12 months
- Moderate to high level of current depressive symptoms (Inventory of Depressive Symptomatology- Self Report, IDS-SR ≥ 21)
- At least one adequate trial of ADM during the current episode (defined as: appropriate doses of antidepressant medication for ≥ 4 weeks; or patient’s refusal to use medication in contrast to advise by a psychiatrist)
- Previous psychological treatment during the current episode (defined as: ≥ 10 sessions of CBT or IPT; or < 10 sessions if discontinued because of patient’s withdrawal)

Exclusion criteria:

- Current psychotic symptoms
- Bipolar disorder according to DSM-IV
- Current alcohol or drugs dependence according to DSM-IV
- Recent electro convulsive therapy (<3 months)
- Current somatic disorder (partly) explaining depressive symptoms
- Physical-, language-, cognitive-, or intellectual impairments which interfere with participation in MBCT or assessments
- Previous MBCT training

### Procedure

**Assessment of eligibility, baseline measures and randomization**

All patients who indicated their interest in the study are invited for a research interview. Written informed consent is obtained during this first appointment. To assess eligibility and to classify comorbid psychiatric disorders, participants are interviewed by a research psychologist or psychiatrists in training with the depression, anxiety, psychosis and addiction modules of the Mini International Neuropsychiatric Interview (MINI; [25]). In addition, the following disorder-related characteristics are assessed: duration of current major depressive episode, number of psychological treatment sessions (i.e. CBT and/or IPT) during current major depressive episode, duration and doses of pharmacological treatments during the current depressive episode, number of depressive episodes during lifetime and age of onset. On the base of these details the research assistant fills out the Dutch Measure for quantification of Treatment Resistant Depression (DM-TRD, [26] [27]). After completion of the baseline questionnaires, a computer program is used to randomly allocate patients to either MBCT + TAU or TAU. A minimization strategy is used for severity of symptoms (IDS-SR ≥21 and ≤31 vs. > 31 and ≤ 39 vs. > 39) and duration of major depressive episode (1–2 years vs. > 2 years). If the period between randomization and start of the MBCT training exceeds four weeks in the MBCT + TAU condition, a repeated baseline assessment takes place.

### Follow-up assessments

The first follow-up measure takes place after the MBCT training or the TAU-period. The MINI depression module is administered face-to-face or by phone and participants fill out the questionnaires either online or on paper. Participants are asked about changes in pharmacological or psychological treatment during the study period. Also participants deciding to discontinue MBCT are asked to participate in follow-up measures. In all MBCT completers (MBCT + TAU and participants of the TAU group who followed the MBCT after the TAU-period), the same follow-up measures are administered at three and six months after end of treatment. Due to logistic reasons it is not possible to keep assessors blind. To assess the inter-rater reliability of the MINI, all diagnostic interviews are audio taped and a randomly selected sample will be reassessed by an independent rater. For an overview of all measurement assessments see Table 1.

**Table 1:** Overview of measures and corresponding measurement time points

Measure	Target concept	Baseline	FU1	FU2	FU3	FU4
IDS-SR	Depressive symptoms	●	●	●	●	●
MINI-depr. module	Diagnosis of major depression	●	●	●	●	●
WHOQOL-BREF	Quality of life	●	●	●	●	●
RRS-EXT	Rumination	●	●	●	●	●
FFMQ	Mindfulness skills	●	●	●	●	●
SCS	Self-compassion	●	●	●	●	●
MINI interview	Comorbid psychiatric disorders	●				
DM-TRD	Treatment-resistance	●				
CTQ	Childhood trauma	●				

*FU1:* 2 weeks post intervention (MBCT + TAU)/8-12 weeks post baseline (TAU), *FU2:* 3 months post intervention (MBCT+ TAU)/2 weeks post intervention (TAU), *FU3:* 6 months post intervention (MBCT + TAU)/3 months post intervention(TAU), *FU4:* 6 months post intervention (TAU)

### Interventions

**MBCT + TAU**

The MBCT consists of eight weekly sessions of 2.5 h in groups of 8–15 patients and one additional day of silent practice between the 5^th^ and 7^th^ session. Participants are asked to do daily homework assignments. MBCT combines mindfulness meditation techniques, as sitting meditation and the body scan with elements of cognitive therapy such as psycho education and the identification of automatic negative thoughts. The MBCT manual described by Segal, Williams and Teasdale [13] is used. Participants are enrolled in regular MBCT trainings provided at the Radboudumc Centre for Mindfulness and Pro Persona for (remitted) depressed patients. This means that the training groups not only consist of study participants but also of regular patients with a wide range of depressive symptoms.

MBCT trainings are taught by experienced mindfulness trainers who completed a 2-year post-graduate teacher training program at the Radboudumc Centre for Mindfulness. With this, they all meet the training criteria described in the MBCT good practice guidelines (UK Mindfulness-Based Teacher Trainer Network, 2011). Two MBCT sessions of each trainer are videotaped to verify adherence to the MBCT protocol. In addition to MBCT the participants receive TAU.

### TAU

TAU may consist of any treatment the participant or current mental health care specialist sees as necessary. Participants are encouraged to continue treatment they followed prior to enrolling in the study. The number of treatment sessions and use of antidepressant medication are recorded.

### Measurements

#### Primary outcome measure

### Depressive symptoms

The primary outcome measure is the level of depressive symptoms measured with the Inventory of Depressive Symptomatology Self-Report (IDS-SR). The IDS-SR is a 30-item self report questionnaire and has good psychometric properties [28]. The IDS-SR has previously been used in research on MBCT in depression and has been shown to be sensitive to change [17].

#### Secondary outcome measures

### Remission rates

Remission from depression according to DSM-IV criteria is assessed with the major depression module of the Mini International Neuropsychiatric Interview (MINI; [25]).

### Quality of life

Perceived quality of life is measured using the 26 -item World Health Organization Quality of Life scale (WHOQOL-BREF; [29]). Psychometric properties were tested in a sample of Dutch psychiatric outpatients and are considered to be good [30].

### Rumination

Rumination is measured with the extended version of the Ruminative Response Scale (RRS-EXT [31, 32]. The RRS-EXT distinguishes between a more reflective way of thinking (i.e. reflective pondering) and a more maladaptive way of thinking about symptoms (i.e. brooding) [33].

### Mindfulness skills

Mindfulness skills are measured with the Five Facets Mindfulness Questionnaire (FFMQ. [34]. The FFMQ consists of 39 items which are divided in five subscales: observing, describing, acting with awareness, non-judging of inner experience and non-reactivity of inner experience. The Dutch version of the FFMQ has good psychometric properties [35, 36] and has previously been used in research on mindfulness skills in bipolar disorder [37].

### Self-compassion

Self-compassion is assessed with the Self Compassion Scale (SCS; [38, 39]). The SCS is an 26-item self report measure and consists of six components: self-kindness, self-judgment, common humanity, isolation, mindfulness, and over-identification. The scale has good psychometric properties and high scores on the SCS are related to psychological well-being [39].

#### Possibly moderating variables

### Treatment-resistance

The Dutch Measure for quantification of Treatment Resistant Depression (DM-TRD; [26, 27]) is used to investigate treatment-resistance of the current depressive episode. With the DM-TRD several dimensions of treatment-resistance are scored by the clinician (duration, symptom severity, functional impairment, comorbid anxiety symptoms, prosocial stressors, treatment failures of pharmacological and psychological treatment) which leads to a total score ranging from 2–26.

### Childhood trauma

To assess the experience of traumatic childhood events, participants fill out the Childhood Trauma Questionnaire (CTQ-SF; [40]). This 28-item questionnaire measures traumatic experiences and maltreatment during childhood. The CTQ-SF has previous been validated in a Dutch clinical sample [41].

### Data handling

All study-related information is stored in secure folders with limited access. Paper-based data collection forms only contain participant numbers to maintain participant confidentiality and are stored in a locked cabinet in an area with limited access. Electronic data files are password protected. The list linking participant number and personal information is stored in a separated password protected file. Paper-based data entry will be double checked. Data will be stored for 15 years after the end of inclusion. The study has low to negligibly risks therefore no data monitoring committee is assigned. Only the first author and principal investigators or persons assigned by them will have access to the final data set. Authors of the final trial report will have made substantial contribution to the design, conduction, interpretation and reporting of the trial. We will not use professional writers. Study results will be presented via publications and presentations; study participants, funders and involved clinicians will receive a summary of the study results.

### Statistical analyses

**Power analysis and sample size**

A power analysis was executed based on the findings of van Aalderen et al. [20]. Van Aalderen and colleagues [20] reported an effect size of 0.53 (Cohen’s *d*) in a sample of 69 currently depressed patients. Based on an alpha of 0.05 and power of 80 %, estimating the correlation between baseline and first follow-up measurements to be 0.5 and intending to use an ANCOVA controlling for baseline levels of depression, a needed sample size of 43 per condition was calculated. Expecting a drop-out of 8 patients, a total of 94 participants will be recruited.

### Analyses plan

The MBCT + TAU and TAU conditions will be compared on baseline sample characteristics to ensure that all possible meaningful characteristics were evenly distributed across the conditions. To examine treatment outcome, we will use both intention-to-treat and per-protocol samples. Participants are considered completers of the MBCT if they attend at least four sessions of MBCT. To examine whether effects for participants experiencing a depressive episode for one to two years and participants meeting criteria for chronic depression according to the DSM-IV (≥2 years) are different, the primary and secondary analyses will be repeated in these subgroups.

### Primary analysis

The effect of condition (MBCT + TAU vs. TAU) on the primary outcome measure (depressive symptoms) will be examined using general linear models. Condition and baseline score of depressive symptoms will be included as independent variables, depressive symptoms at FU1 as the dependent variable. Because MBCT is delivered in mixed groups consisting of regular patients and a few study participants and previous studies did not yield an effect of therapy group [20], we will not incorporate a random group effect.

### Secondary analyses

Effects on quality of life, rumination, mindfulness skills, and self-compassion will also be investigated using general linear models with the same independent variable as in the primary analysis. To investigate effects on remission rates a χ^2^ test will be performed. Moderating effects of level of treatment-resistance and childhood trauma will be investigated by including an interaction effect (condition*moderator) in the models. Furthermore, exploratory analyses on rumination, mindfulness skills and self-compassion as possible underlying mechanisms of change will be conducted. Consolidation and moderation of treatment effect will be investigated in the whole sample of MBCT completers (MBCT + TAU and TAU) using end-of-treatment assessments and follow-up assessments at three and six months after end of treatment.

## Discussion

Despite the range of currently available treatments options such as antidepressant medication and cognitive behavioral or interpersonal therapy, a substantial number of currently depressed patients do not recover [10]. Chronic, treatment-resistant depression is associated with a high personal as well as societal burden [4, 5]. Additional treatment options are required, to further reduce depressive symptoms and to improve quality of life in this population.

In previous pilot studies, MBCT appeared to be a promising treatment option for patients with chronic depression. Besides incorporating effective elements of cognitive behavioral therapy, MBCT focuses on acknowledging negative emotions and thoughts and disengaging from maladaptive automatic cognitive and behavioral patterns elicited by them. As chronic, treatment-resistant depression is characterized by perseverance of symptoms and a long treatment history, maladaptive automatic thinking patterns are likely to have become habitual. Therefore MBCT might be a very valuable additional treatment for this group. In the current study we will build upon the previous pilot findings by studying the effect of MBCT on depressive symptoms in a large chronic, treatment-resistant sample in a randomized controlled trial.

Strauss and colleagues [19] argue that MBCT might be as effective as cognitive behavioral therapy in reducing depressive symptoms in currently depressed patients. The results of the current study will provide an indication of the effectiveness of MBCT over and above available pharmacological and psychological treatments such as CBT and IPT, in patients who previously received these types of psychological treatment.

In addition to the effects of MBCT on depressive symptoms, we will investigate if MBCT leads to less rumination and higher quality of life in this patient population. Moreover, this trial will inform clinicians and researchers if chronic, treatment-resistant depressed patients are able to learn mindfulness skills and to develop more self-compassion by following a standard MBCT training. This large trial will also give more detailed information about the acceptability and effect sizes of MBCT in this target group.

The ecological validity of the study will be high, as participants will be enrolled in standard Dutch MBCT courses offered at mental health care institutions. If the conclusion will be that MBCT is effective in reducing depressive symptoms in chronic, treatment-resistant depression, this study could make a significant impact on treatment options for this severely depressed target group.

## Abbreviations

ADM: Antidepressant medication
CBT: Cognitive behavioral therapy
CTQ-SF: Childhood Trauma Questionnaire-short form
DM-TRD: Dutch measure of treatment resistant depression
DSM-IV: Diagnostic and statistical manual of mental disorders- 4^th^ edition
FFMQ: Five facet mindfulness questionnaire
IDS-SR: Inventory of Depressive Symptomatology- Self Report
IPT: Interpersonal therapy
MBCT: Mindfulness-based Cognitive Therapy
MINI: Mini International Neuropsychiatric Interview
RRS-EXT: Ruminative response scale extended version
SCS: Self compassion scale
TAU: Treatment as usual
WHOQoL-BREF: World health organization quality of life scale
